# Effect of species, breed, and age on bacterial load in bovine and bubaline semen

**DOI:** 10.14202/vetworld.2015.461-466

**Published:** 2015-04-09

**Authors:** Chandrahas Sannat, Ajit Nair, S. B. Sahu, S. A. Sahasrabudhe, Ashish Kumar, Amit Kumar Gupta, R. K. Shende

**Affiliations:** 1 Department of Veterinary Microbiology, College of Veterinary Science & A.H., Chhattisgarh Kamdhenu Vishwavidayalaya, Anjora, Durg Chhattisgarh, India; 2 Central Semen Station, Livestock Development Department, Government of Chhattisgarh, Anjora, Durg, Chhattisgarh, India

**Keywords:** age group, bacterial load, breed, buffalo bulls, cow bulls, semen

## Abstract

**Aim:**

The present study was conducted to investigate the effect of species, breed and age on bacterial load in fresh and frozen semen of Cattle and Buffalo bull.

**Materials and Methods:**

Present study covered 56 cow and 10 buffalo bulls stationed at Central Semen Station Anjora, Durg (Chhattisgarh). Impact of breeds on bacterial load in semen was assessed using six breeds of cattle *viz*. Sahiwal, Gir, Red Sindhi, Tharparkar, Jersey and Holstein Friesian (HF) cross. Cow bulls were categorized into four different groups based on their age (<4 years, 4-5 years, 5-6 years and > 6 years) to study variation among age groups. Bacterial load was measured in fresh and frozen semen samples from these bulls using the standard plate count (SPC) method and count was expressed as colony forming unit (CFU) per ml of semen.

**Results:**

Higher bacterial load was reported in fresh (2.36 × 10^4^ ± 1943 CFU/ml) and frozen (1.00 × 10 ± 90 CFU/ml) semen of cow bulls as compared to buffalo bulls (1.95 × 10^4^ ± 2882 and 7.75 × 10^2^ ± 160 CFU/ml in fresh and frozen semen, respectively). Jersey bull showed significantly higher bacterial count (p < 0.05) both in fresh (4.07 × 10^4^ ± 13927 CFU/ml) and frozen (1.92 × 10^3^ ± 178 CFU/ml) semen followed by HF cross, Sahiwal, Gir, Red Sindhi and Tharparkar bull. Bulls aged < 4 years and more than 6 years yielded increased bacterial load in their semen. Although a minor variation was reported between species and among age groups, no significant differences were measured.

**Conclusion:**

Bacterial load in semen did not differ significantly between species and age groups; however significant variation was reported among different breeds. Bulls of Jersey breed showed significantly higher bacterial load in semen as compared to the crossbred and indigenous bull.

## Introduction

Cattle and buffalo play an important role in rural livestock production by providing milk, meat, and work draft forces. About 80% of total cattle population consisted of the low producing nondescript animal. Breeding policy has been set up for conservation and development of these animals by Government of India as well respective states of India. Though breeding policy varies with region and locality specific features such as climate condition, feed and fodder availability, and livestock management practices, two important strategies for breed improvement included upgrading with indigenous breeds and crossbreeding with exotic breeds. Genetic improvement in these animals is carried out by artificial insemination technique using cryopreserved semen of potential cattle and buffalo bulls. Apart from providing desired genetic characteristics, semen could act as a vehicle for the wide range of undesirable pathogens [1]. Organisms can be acquired either from infection in animals, preputial sheath or from the environment during collection, processing or packaging of semen. The bacterial contaminants of semen have been a major concern for most semen production laboratories as it adversely affects the semen quality [2] and hence the subsequent fertility [3]. Hence, the success of AI program depends on quality semen production in the laboratory.

To ensure clean semen production routine, surveillance of bacterial load in semen is needed. Lot of workers have worked on bacteriological analysis of semen both of cow bulls [4-7] and buffalo bulls [5,8,9] and also reported minor variation among species. Based on breeding policies of respective states, different breeds of cattle and buffalo are used for genetic improvement. Bacterial load in semen may vary among individual bulls of similar breed or between breeds also [4]. Although, bacterial load in semen was being evaluated in different cattle breeds, i.e., Holstein Friesian (HF) [10], crossbred cattle [6], Jersey and Guernsey [4], Hereford [11], Sahiwal [12] and Gir [5], none of the report presented comparative analysis between breeds. Apart from this, bulls used for semen collection in semen station belong to different age group and as per norms of minimum standard protocol for production of bovine semen, semen collection may continue from bulls up to the maximum age of 10 years [13]. Previously, attempt was already made to establish a correlation between age of the bull and microbial load in semen [11].

Hence, a better knowledge of the effect of species, breed, and age on semen microbes can help the AI industry to adapt a standard management of bulls to improve semen output. Taking above facts into consideration, an attempt was made to investigate the influence of species, breed, and various age groups on bacterial load in fresh and frozen semen of cow and buffalo bull stationed at Central Semen Station, Anjora, Durg (Chhattisgarh).

## Materials and Methods

### Ethical approval

Ethical approval was not necessary. On routine semen collection days, samples were taken from animals. Proper ethical considerations related to animal handling were observed and ensuring not to cause any injury during sampling.

### Bulls under study

A total of 56 cow and 10 buffalo bulls stationed at central semen station Anjora, Durg (Chhattisgarh) were taken into study. Due to availability of only one breed and less population size in various age groups of buffalo bull, investigation regarding variation between breeds, and age groups was not conducted for buffalo bulls and only cow bulls were analyzed for the same. Semen samples collected from six breeds of cattle, i.e. Sahiwal, Gir, Red Sindhi, Tharparkar, HF cross and Jersey breed were evaluated for bacterial load to assess the variation among breeds. Semen collection starts at age when bull reaches sexual maturity and continued for a maximum of 5-6 years [13]. As per the availability of bulls of different age groups, bulls under study were categorized into four groups *viz*. Below 4 years, 4-5 years, 5-6 years and above 6 years. During the course of study, all bulls were being maintained under similar feeding and managemental practices.

### Screening of bulls for communicable diseases

All bulls were being routinely screened (every ½ yearly) for major contagious diseases before their entry to semen station. As per OIE guidelines and minimum standard protocol for production of bovine semen [13], breeding bulls were tested for bacterial diseases namely tuberculosis, paratuberculosis, brucellosis and genital campylobacteriosis by an accredited agency i.e. Western Region Disease Diagnostic Laboratory, Pune. Bulls under the present study were found negative for above communicable diseases.

### Collection and processing of semen samples

Semen samples were collected by means of sterile artificial vagina using routine collection technique [14]. Strict aseptic measures were practiced during collection and handling of semen samples. Physical characteristics of fresh ejaculates are summarized in Table-1. Fresh semen samples were processed immediately for bacteriological examination within 1 h after collection. Frozen semen straws were prepared using standard processing techniques. Conventional antibiotics, i.e. streptomycin sulfate (100 mg/ml) and penicillin (100 IU/ml) were added in the semen extender to overcome bacterial growth [15].

**Table-1:** Physical characteristics of bull semen.

Breed	Number of bulls	Number of ejaculates tested	Color	Volume (ml)	Sperm concentration (millions/ml)	Progressive motility (%)
Sahiwal	13	52	Milky creamy and creamy	4.5±0.29	1252±71	73±2.1
Gir	8	32	Milky creamy, creamy, and slightly yellowish	5.3±0.25	1268±85	71±2.1
Red Sindhi	9	36	Milky creamy and creamy	5.4±0.34	1359±134	73±1.3
Tharparkar	8	32	Milky creamy to creamy	4.7±0.31	1449±115	74±0.9
Jersey	7	28	milky creamy to creamy and watery in few cases	4.9±0.23	1066±73	75±2.3
HF cross	7	28	Milky creamy to creamy and watery in few cases	7.6±0.33	1008±92	74±1
Murrah	10	40	Milky creamy to creamy	3.7±0.27	1051±63	79±1.7

HF=Holstein Friesian

### Determination of bacterial load in semen

The bacterial load in the semen samples was measured using standard plate count (SPC) method [16]. Culture media and reagents of HiMedia were used throughout the study.

### Fresh semen

Tenfold serial dilution of the semen sample (1:10, 1:100, 1:1000 and 1:10000) was made in sterile nutrient broth. Inoculums size of 0.05 ml from each dilution was mixed thoroughly with molten SPC agar (previously held in the water bath at 50°C) and poured into sterile petri dish plates. Separate plates were used with each dilution and two SPC agar plates were taken for a single batch of semen. Agar was allowed to set and then incubated at 37°C for 72 h. Colonies per plate were read and counted with the help of colony counter. Colony forming unit (CFU) per ml of sample was calculated using the formula: CFU/ml of sample = No. of CFU’s × dilution × 2.

### Frozen semen

Frozen semen straws were thawed by placing it in the water bath at 37°C for 30 s. After drying, straws were disinfected by 70% ethanol and both sides of frozen semen straw has been broken using a sterile scissor, in order to take a drop of semen after neglecting the first drop. For each sample, four numbers of French mini straws (0.25 ml) were thawed, to obtain inoculums size of 0.5 ml. Then, samples were serially diluted in sterile nutrient broth and processed for bacterial load as per the technique mentioned above for fresh semen.

### Data recording and statistical analysis

Data were expressed as means (±standard error of the mean) CFU/ml of semen and analyzed by applying general linear model for factorial experiments using SPSS computer software package (Version 16.0.0.247^©^2007). Duncan’s multiple range tests was done to make specific treatment comparisons for values that were found significant by ANOVA.

## Results

### Species wise variation of bacterial load

Present study reported bacterial load of 2.36 × 10^4^ ± 1943 and 1.95 × 10^4^ ± 2882 CFU/ml in fresh semen of cattle and buffalo, respectively. However, bacterial load observed in frozen semen of cattle and buffalo were 1.00 × 10^3^ ± 90 and 7.75 × 10^2^ ± 160 CFU/ml, respectively (Table-2). Though present investigation reported lower bacterial load in fresh and frozen semen of buffalo bull as compared to cow bull, no significant variation was observed between species (p > 0.05).

**Table-2:** Species wise variation of bacterial load in semen.

Species	Number of bulls	Number of ejaculate tested	CFU/ml (mean±SEM)	

			Fresh semen	Frozen semen
Cattle	56	224	2.36×10^4^±1943^a^	1.00×10^3^±90^a^
Buffalo	10	40	1.95×10^4^±2882^a^	7.75×10^2^±160^a^

Note: Values having similar superscript in a column differ non-significantly (p>0.05), SEM=Standard error of mean, CFU=Colony forming unit

### Breed wise variation of bacterial load

Bacterial load observed in semen from different breeds of cattle is shown in Table-3. Significantly higher bacterial load was reported in fresh (4.07 × 10^4^ ± 13927 CFU/ml) as well frozen (1.92 × 10^3^ ± 178 CFU/ml) semen of Jersey bull followed by HF cross, Sahiwal, Gir, Red sindhi, Tharparkar in case of fresh semen and HF cross, Gir, Sahiwal, Red sindhi and Tharparkar in case of frozen semen. Bacterial load in fresh semen of Jersey bull differed highly significantly from Tharparkar and Red Sindhi (p < 0.01) and significantly with Sahiwal and Gir bull (p < 0.05) while no significant variation was seen between Jersey and HF cross. However, bacterial load in frozen semen of Jersey bull differed highly significantly (p < 0.01) from all other breeds under study. In frozen semen, Gir showed higher bacterial load as compared to Sahiwal bull, but did not differ significantly (p > 0.05).

**Table-3:** Breed wise variation of bacterial load in cow bull semen.

Breed	Number of bulls	Number of ejaculates tested	CFU/ml (mean±SEM)	

			Fresh semen	Frozen semen
Sahiwal	13	52	2.36×10^4^±2604^ab^	9.61×10^2^±138^a^
Gir	8	32	2.34×10^4^±2161^ab^	1.09×10^3^±240^a^
Tharparkar	9	36	1.75×10^4^±3975^a^	6.11×10^2^±172^a^
Red sindhi	8	32	1.90×10^4^±5042^a^	6.56×10^2^±226^a^
Jersey	7	28	4.07×10^4^±13927^b^	1.92×10^3^±178^b^
HF cross	7	28	2.42×10^4^±3023^ab^	1.10×10^3^±224^a^

Note: Values with different superscript in a column differ significantly, SEM=Standard error of mean, HF=Holstein Friesian, CFU=Colony forming unit

### Influence of age on bacterial load

Non-significant differences (p > 0.05) of bacterial load was noted between different age groups. However, comparatively higher bacterial load was observed in semen of young bulls (aged below 4 years) followed by older bulls (above 6 years). Bulls aged 4-5 and 5-6 years yielded relatively lower bacterial load in their semen (Table-4).

**Table-4:** Effect of age of cow bull on bacterial load in their semen.

Age group	Number of bulls	Number of ejaculates tested	CFU/ml (mean±SEM)	

			Fresh semen	Frozen semen
<4 years	13	26	2.82×10^4^±4006^a^	1.21×10^3^±180^a^
4-5 years	19	38	2.35×10^4^±5264^a^	7.36×10^2^±124^a^
5-6 years	12	24	1.92×10^4^±2805^a^	1.04×10^3^±253^a^
More than 6 years	12	24	2.45×10^4^±3438^a^	1.14×10^2^±177^a^

Note: Values having similar superscript in a column differ non-significantly (p>0.05), CFU=Colony forming unit, SEM=Standard error of mean

## Discussion

### Species wise variation of bacterial load

Variation of bacterial load in semen of cattle and buffalo was supported by Hasan *et al*.[12] and present finding may be compared with observation of Jaisal *et al*. [5], who also reported increased bacterial count (5 × 10^3^-5.6 × 10^3^ CFU/ml) in fresh semen of cow bull than buffalo bull (4.1 × 10^3^-1.8 × 10^4^ CFU/ml). Likewise, Gunsalus *et al*. [17], Meredith [18], Brown *et al*. [11], Miller and Salisbury [19] and Almquist *et al*. [4] also observed higher bacterial load of 2.2 × 10^4^, 36.5 × 10^4^, 2.31 × 10^4^, 3 × 10^5^-3 × 10^7^ and 1 × 10^2^-3 × 10^7^ CFU/ml respectively, in fresh semen of cow bull. Lower bacterial load reported in frozen semen doses were due to the effect of added antibiotics [15]; however in a report [20] it was mentioned that 13% of isolated bacteria from semen were resistant to penicillin and streptomycin, the most common antibiotic combination used in semen diluents.

Bacterial load reported in frozen semen of cow bull in present finding is in agreement with the findings of Kumar *et al*. [21], Patel *et al*.[6] and Wierzbowski *et al*. [22] who also reported similar bacterial load of 1.1 × 10^2^, 1.26–5.9 × 10^4^ and 1.1 × 10^3^ CFU/ml, respectively in frozen semen. Likewise present observation, similar bacterial count of 0.73 × 10^2^ CFU/ml [8]; 1.0-5.0 × 10^2^ CFU/ml [5] and 0.41 × 10^2^ CFU/ml [7] were reported in frozen semen of buffalo bull too. In contrast to present finding, Rathnamma *et al*.[10] reported higher bacterial load (5.05-171.4 × 10^3^ CFU/ml) in frozen semen of buffalo bull than that of cow bull (0.81-39 × 10^3^ CFU/ml). In a study [23], higher incidence of microbial population were recorded in preputial cavity of breeding cow bull as compared to buffalo bull, which might account for higher bacterial load in semen of cow bull than buffalo bull. Furthermore, lower bacterial count in buffalo semen may be correlated with higher semen mass activity, higher individual sperm motility, and higher post-thaw motility of buffalo semen as compared to cattle semen [24].

### Breed wise variation of bacterial load

Present study reported significantly higher bacterial count in exotic and crossbred cattle than indigenous breeds. Different coworkers have evaluated bacterial load in different breeds, but none of them have analyzed variation between breeds. In a comparative study between three breeds, marked variation of bacterial load was reported between semen samples from different bulls [4]; in which Guernsey, HF and Jersey bulls yielded bacterial load of 26 × 10^4^, 13 × 10^4^ and 5.3 × 10^4^ CFU per ml of fresh semen, respectively. As per present investigation, higher count of 1 × 10^3^-22 × 10^6^ CFU/ml [17] and 3 × 10^5^-3 × 10^7^ CFU/ml [19] was reported in crossbred cattle. More or less similar results were reported in fresh semen of HF [10], Gir [5] and Sahiwal semen [12].

Bacterial load in frozen semen of HF cross may be compared with observation of Wierzbowski *et al*. [22] and Patel *et al*. [6] who reported bacterial count of 1.1 × 10^3^ and 1.9 × 10^3^ CFU/ml, respectively. In contrast, less bacterial load of 1.1 × 10^2^ and 1.62 × 10^2^ CFU/ml was reported by Kumar *et al*. [21] and Patel and Patel. [25], respectively in frozen semen of crossbred cattle and 0.89 × 10^2^ CFU/ml by Hassan *et al*. [12] in Sahiwal bull. In another group of study [6,10,25], lower bacterial load was reported in frozen semen of HF bull.

Wide variation of bacterial count in semen samples between different bulls and breeds might be due to day-to-day- fluctuation which occurred in the bacterial content of semen collected from apparently healthy animals. In a study [11], it was reported that concentration of natural inhibitors in seminal plasma varies from bull to bull and among breeds and higher concentration of inhibitors was reported in seminal plasma of HF than Hereford cattle. Significant differences of bacterial load in semen of different breeds might be correlated with the level of cytokine expression in cells of different breeds leading to the inherent resistance against infection [26]. The preputial orifice of bull may be a major source of the different types of bacteria found in soil, bedding, and manure [27]. It was reported that Gir bulls were being predisposed to prolapse of the preputial sheath thus exposed to infection, which might accounts for higher bacterial count in semen [28]. In present investigation, it was observed that newly purchased young bulls (08 out of 14 bulls) in Sahiwal group showed increased bacterial load in their semen, which might account for overall higher load in Sahiwal bull as compared to other indigenous breeds. Higher bacterial load in exotic and crossbred cattle than indigenous breeds could be explained. Variations in bacterial load are caused by several factors such as cleanliness, ambient temperature, relative humidity, and photoperiod [29]. The ambient temperatures of 35-40°C with a relative humidity of 35-45% reduced semen quality significantly, as bacterial population in the semen grow best at 20-40°C [30]. Durg, being a tropical and sub-humid region of Chhattisgarh has a long summer period and short winter with average ambient temperature ranging from 30°C to 45°C [31], which favored bacterial growth. These environmental conditions are not suitable for exotic breeds from temperate regions and, therefore, can adversely affect reproductive efficiency of Jersey bulls. Hence, these adverse conditions might lead stress in Jersey bulls resulting reduction in resistance against infection, thus favoring the proliferation of the bacterial population. However, crossbred cattle (HF cross) are well-adapted to the local hot and humid climatic conditions [32]. Decreases in semen quality were less severe, occurred later, and recovered more rapidly in crossbred bulls than in exotic bulls exposed to high ambient temperatures [33]. Thus, a continuous evaluation of their semen quality is required to achieve higher non-return rates and also to keep the crossbreeding program economically viable. On other hand, indigenous breeds are well known for their heat tolerance, resistance to various diseases, adaptability to atmospheric changes [34], these facts were supported by Tarate *et al*. [35] and Katariya [36], who observed higher somatic cell count in milk of crossbred cattle as compared to indigenous breed.

### Influence of age on bacterial load

Comparatively, higher bacterial load in semen of young and older bulls are supported by Brown *et al*. [11] and Almquist *et al*. [4], respectively. Bacterial load observed by Brown *et al*. [11] for bulls aged 2, 3, 5, 6, 8 and 10 years were 5.3 × 10^4^, 4.5 × 10^3^, 8.1 × 10^3^, 3.7 × 10^3^, 4.4 × 10^3^ and 5.7 × 10^3^ CFU/ml respectively. Relatively higher bacterial load in young and old aged bulls could be attributed to poor immune system of those animals, making the bulls more prone to infection. On contrary, in an investigation [37] higher bacterial load was reported in middle-aged camels (9-13 years) than young (4-8 years) and old aged (14-18 years) animals.

## Conclusion

Bacterial load was evaluated in semen of six breeds of cow and one breed of buffalo bull belonging to various age groups and a comparative analysis was made between species, breeds, and age groups. Despite of variation of bacterial load in bovine and bubaline semen, no significant differences were measured between species. However, bacterial load between breeds varied significantly and significantly higher bacterial load was observed in semen of Jersey bull followed by cross-bred animals and indigenous cattle breeds. Higher bacterial count was reported in young and old bulls, but differences measured were non-significant.

## Authors’ Contributions

CS and AN designed the experiment. Sample collection and preparation of frozen semen straws was performed by SBS, CS, and AN under supervision of SAS. Sample processing, overall experiment and statistical analysis were done by CS, AK, AKG, and RKS under supervision of SAS. Manuscript preparation was reviewed and edited by all authors. All authors read and approved the final manuscript.
